# Does parental permissiveness toward cigarette smoking and alcohol use influence illicit drug use among adolescents? A longitudinal study in seven European countries

**DOI:** 10.1007/s00127-021-02118-5

**Published:** 2021-06-13

**Authors:** Emina Mehanović, Federica Vigna-Taglianti, Fabrizio Faggiano, Maria Rosaria Galanti, The EU-Dap Study Group, Barbara Zunino, Federica Vigna-Taglianti, Gian Luca Cuomo, Serena Vadrucci, Silena Salmaso, Karl Bohrn, Sebastian Bohrn, Erwin Coppens, Yannick Weyts, Peer van der Kreeft, Johan Jongbloet, Juan Carlos Melero, Tatiana Perez, Laura Varona, Oihana Rementeria, Gudrun Wiborg, Maro Vassara, Maria Kyriakidou, Gabriela Terzopoulou, Sara Sanchez, Charlotte Jansson, Maria Rosaria Galanti, Fabrizio Faggiano, Leila Fabiani, Maria Scatigna

**Affiliations:** 1 Department of Clinical and Biological Sciences, University of Torino, Orbassano, Torino, Italy; 2 Piedmont Centre for Drug Addiction Epidemiology, ASL TO3, Grugliasco, Torino, Italy; 3 Department of Translational Medicine, Avogadro University, Novara, Italy; 4 Department of Global Public Health, Karolinska Institutet, Stockholm, Sweden; 5 Centre for Epidemiology and Community Health, Stockholm County Council, Stockholm, Sweden

**Keywords:** Parental permissiveness, Illicit drug use, Adolescents, Gender, Longitudinal study, Mediators

## Abstract

**Purpose:**

Adolescents’ perceptions of parental norms may influence their substance use. The relationship between parental norms toward cigarette and alcohol use, and the use of illicit substances among their adolescent children is not sufficiently investigated. The purpose of this study was to analyze this relationship, including gender differences, using longitudinal data from a large population-based study.

**Methods:**

The present study analyzed longitudinal data from 3171 12- to 14-year-old students in 7 European countries allocated to the control arm of the European Drug Addiction Prevention trial. The impact of parental permissiveness toward cigarettes and alcohol use reported by the students at baseline on illicit drug use at 6-month follow-up was analyzed through multilevel logistic regression models, stratified by gender. Whether adolescents’ own use of cigarette and alcohol mediated the association between parental norms and illicit drug use was tested through mediation models.

**Results:**

Parental permissive norms toward cigarette smoking and alcohol use at baseline predicted adolescents’ illicit drug use at follow-up. The association was stronger among boys than among girls and was mediated by adolescents’ own cigarette and alcohol use.

**Conclusion:**

Perceived parental permissiveness toward the use of legal drugs predicted adolescents’ use of illicit drugs, especially among boys. Parents should be made aware of the importance of norm setting, and supported in conveying clear messages of disapproval of all substances.

## Introduction

Illicit drug use among European adolescents is a serious public health concern. According to the Health Behaviour in School-aged Children (HBSC) estimates, the prevalence of lifetime and recent cannabis use among 15-year-old European adolescents is 13% and 7%, respectively [1]. According to the European School Survey Project on Alcohol and Other Drugs (ESPAD) survey, the prevalence of lifetime illicit drug use among 15- to 16-year-old students is 17% [2].

Parental behaviors, expectations, attitudes and norms play an important role in shaping adolescent substance use behaviors. It is well documented that parental permissive attitudes toward cigarette, alcohol and marijuana increase adolescent's risk of use of the same substance, whereas parental disapproval exerts a protective effect [3–18].

Parental behaviors and norms may generalize across the use of different substances. Negative parental norms toward all kind of substances, both licit and illicit, reduce the risk of use in their offsprings [19–27]. However, the question whether parental norms toward a given substance affect the use of other substances has been addressed in very few studies. Parental permissiveness toward alcohol use was associated with adolescents' engagement in cigarette and marijuana use in the studies by Voisine et al. (2008) and Harakeh et al. (2012) [16, 28]. In a recent study conducted in the Netherlands [29], parental strict rules regarding alcohol drinking were not only associated with reduced alcohol consumption but subsequently also with tobacco and cannabis use. Some studies reported that parental permissive attitudes toward gambling, delinquency and antisocial behavior, were also associated with the risk of cigarette, alcohol and cannabis use [20, 30, 31], suggesting that adolescents' substance use may be influenced by parental attitudes toward other risk behaviors.

The influence of parental lenient or disapproving attitudes on adolescent substance use did not differ substantially between boys and girls in cross-sectional studies [5, 16, 19, 24]. However, a longitudinal study conducted in Australia found a potential protective effect of parental disapproval on alcohol use to be stronger among boys than among girls [32]. Generally, girls perceive greater parental disapproval of substance use and disapprove substance use themselves to a greater extent compared to boys [5, 16, 24, 32].

The studies conducted so far did not present consistent results on gender differences in the association between parental permissiveness toward legal substance use and offspring’s use of illicit drugs. The purpose of this study is to expand the existing knowledge by analyzing the longitudinal effect of parental permissiveness toward cigarette and alcohol use on the use of illicit drugs among adolescents, exploring gender differences.

## Materials and methods

### Study design and population

The present study analyzed longitudinal data from 3171 students allocated to the control arm of the European Drug Addiction Prevention (EU-Dap) trial (www.eudap.net) and providing answer to the question on past 30 days illicit drug use at 6-month follow-up. The trial took place in seven European countries (Austria, Belgium, Germany, Greece, Italy, Spain and Sweden) and involved a total of 7079 students aged 12–14 years. To analyze the effect of parental norms on pupils’ behaviors independently from the experimental intervention, the current analysis was limited to the control school students.

Using an anonymous code self-generated by the student, 89.8% of baseline responses in the control arm could be linked to their corresponding 6-month follow-up survey data. The study design was described in detail elsewhere [33].

### Data collection

A self-completed anonymous questionnaire was used to collect information about sociodemographic characteristics, school performance, substance use behaviors, knowledge, attitudes, beliefs and risk perceptions, friends’ substance use, skills, parental cigarette use, and perceived parental permissiveness at baseline (October 2004) and at follow-up (May 2005). Most questions were derived or adapted from the Evaluation Instruments Bank of the Exchange on Drug Demand Reduction Action (EDDRA), the online platform of European Monitoring Centre for Drugs and Drug Addiction (EMCDDA) that provides validated instruments for evaluating prevention, treatment, and harm reduction interventions (http://www.emcdda.europa.eu/eib). To preserve anonymous management of the data, the questionnaires were labeled with a 9-digit individual code generated by the student [34]. A general policy of informed consent was not adopted. Each center followed the practice required locally to obtain permission from the corresponding Ethical Boards [33].

### Measures

Adolescent’s cannabis use was investigated by asking “How many times (if any) have you used marijuana or hashish during the last 30 days?” with responses ranging from 0 to 30 and more. Other illicit drug use was investigated by asking “Have you used any of the following drugs during the last 30 days?” Tranquillizers/sedatives (without a doctor’s prescription); LSD or some other hallucinogens; amphetamines; crack; cocaine; relevin (false substance); heroin; ecstasy; GHB; methadone; “magic mushrooms”; ketamine. The question investigated the use of each drug separately. The answers to the items on marijuana or hashish and other illicit drug use were collapsed into a dichotomous indicator of any illicit drug use in the past 30 days.

Individual sociodemographic information included gender, age (based on date of birth) and family composition (living with “both parents”, “one parent”, and “other”).

Recent adolescents’ cigarette smoking and alcohol use were investigated by asking students “How many times (if any) have you smoked cigarettes during the last 30 days?” and “How many times (if any) have you been drunk from drinking alcoholic beverages during the last 30 days?” with responses ranging from 0 to 30 and more.

The smoking behavior of parents was investigated asking the students if their mother and father smoked cigarettes, with possible answers “Smokes daily”, “Smokes sometimes”, “Does not smoke”, “Do not know”, “Do not have or see this person”.

Perceived parental permissiveness toward cigarette smoking was examined through the question “If you wanted to smoke (or already do), do you think your father and mother would allow you to do so?”, with possible responses “Would allow (allows me) to smoke”, “Would not (does not) allow smoking at home”, “Would not (does not) allow smoking at all” and “Do not know”. A similar question was used to investigate perceived parental permissiveness toward alcohol use “If you wanted to drink alcohol (or already do), do you think your father and mother would allow you to do so?”, with possible responses “Would allow (allows me) to drink alcohol”, “Would not (does not) allow drinking at home”, “Would not (does not) allow drinking at all” and “Do not know”. The associations between perceived permissiveness reported in these two questions and the outcome variable were examined both by keeping the answers “would not allow at home” and “would allow” as separate categories and by merging them into one category. A high proportion of students answered “Do not know” (9.9% for parental permissiveness to smoke and 19.0% for parental permissiveness to drink alcohol), so this was used as separate category in the analysis.

### Statistical analysis

For the purpose of the present analyses, a dichotomous outcome variable of any illicit drug use in the past 30 days at 6-month follow-up was used as described above. Only students with both baseline and follow-up information were included in the analyses. In the primary analysis, both users and non-users of illicit drugs at baseline were included, thus the outcome variable at follow-up measured both initiation and continued use. The model was stratified by gender. A secondary analysis was conducted limiting the sample to baseline past 30-day non-users (*n* = 2975) to study the onset of use.

Multilevel mixed-effect logistic regression models with three levels (country, school and student) were fitted to estimate the Prevalence Odds Ratios (APOR) and Risk Ratios (RR), respectively. Adjustments were made for gender, age, family composition and parental cigarette smoking. Parental permissiveness toward cigarette smoking and parental permissiveness toward alcohol use were examined as separate predictors due to collinearity. Mediation analysis was conducted on baseline past 30-day non-users to test the mediating effect of adolescents’ own cigarette and alcohol use at follow-up on the relationship between parental permissiveness and illicit drugs use using the PROCESS macro for SPSS [35]. The mediation effect was tested separately for each mediator adjusting for confounders. The indirect effect and 95% Confidence Interval were obtained through bootstrapping (1000).

There were less than 2.2% missing data in all studied variables. Applying pairwise deletion, the final model of parental permissiveness to smoke cigarettes was run on 3075 students (97.0% of the initial sample), and of parental permissiveness to use alcohol on 3064 students (96.6% of the initial sample). Statistical analyses were carried out using STATA software release 12.0 and SPSS software release 26 [36, 37].

## Results

The prevalence of recent illicit drug use at baseline was 5.8% (3.9% used cannabis and 2.8% used other illicit drugs), and increased at follow-up to 9.3% (7.3% used cannabis and 4.3% used other illicit drugs).

The prevalence of illicit drugs use at follow-up was higher among 14-year-olds than among 12-year-olds (17.1% vs. 4.0%). Adolescents from one-parent families reported higher involvement in illicit drug use compared to those from families composed of two parents (14.1% vs. 8.6%). Adolescents whose parents smoked cigarettes reported higher proportion of illicit drug use at follow-up compared to their peers whose parents did not smoke (11.4% vs. 6.5%). Finally, the prevalence of illicit drug use was much higher among adolescents whose parents were permissive toward cigarettes (21.9% vs. 7.2%) or alcohol (19.4% vs. 6.1%) compared to those whose parents did not permit the use of these substances. The prevalence of illicit drug use was higher among boys than among girls for all analyzed predictors (Table 1).

**Table 1:** Prevalence of illicit drug use at follow-up by gender and baseline indicators

Characteristics	Overall (295/3171)		Boys (195/1627)		Girls (98/1537)	
	*n*/*N*	%	*n*/*N*	%	*n*/*N*	%
Age						
≤ 12	33/836	4.0	19/418	4.6	13/415	3.1
13	28/967	2.9	14/481	2.9	13/483	2.7
≥ 14	234/1368	17.1	162/728	22.3	72/639	11.3
Family composition						
Both parents	216/2510	8.6	150/1297	11.6	66/1209	5.5
One parent	38/269	14.1	22/118	18.6	16/151	10.6
Other	41/387	10.6	23/207	11.1	16/177	9.0
Parental cigarette smoking						
No	90/1383	6.5	60/683	8.8	30/697	4.3
Yes	200/1755	11.4	130/920	14.1	68/831	8.2
Perceived parental permissiveness to smoke cigarettes						
Would not allow at all	169/2338	7.2	106/1184	9.0	63/1149	5.5
Would not allow at home	56/305	18.4	39/160	24.4	16/144	11.1
Would allow	35/160	21.9	24/93	25.8	11/67	16.4
Do not know	31/307	10.1	22/151	14.6	8/155	5.2
Perceived parental permissiveness to use alcohol						
Would not allow at all	114/1862	6.1	69/914	7.6	45/945	4.8
Would not allow at home	62/344	18.0	45/194	23.2	17/150	11.3
Would allow	59/304	19.4	41/167	24.6	16/134	11.9
Do not know	55/590	9.3	35/309	11.3	20/280	7.1

Any use of cannabis, tranquillizers/sedatives (without a doctor’s prescription), LSD or some other hallucinogens, amphetamines, crack, cocaine, relevin (false substance), heroin, ecstasy, GHB, methadone, “magic mushrooms” and ketamine in the past 30 days

The perceived parental permissiveness toward cigarettes and alcohol use increased from baseline to follow-up by 2.6% and 5.5%, respectively. The increment of perceived permissive norms was seen both among boys and girls. A greater proportion of boys than girls had positive perception of parental norms toward cigarettes at baseline (15.9% vs. 13.9%) and toward alcohol drinking in both occasions (22.8% vs. 18.9% at baseline, and 30.2% vs. 22.6% at follow-up).

Controlling for potential confounders (age, gender, family composition and parental smoking), perceived permissive parental norms toward cigarette and alcohol predicted the use of illicit drugs among adolescents at 6-month follow-up (Table 2). Adolescents who perceived that their parents would allow smoking and those who stated their parents would not allow smoking at home had about 2 times higher prevalence of past 30-day illicit drug use at follow-up compared to adolescents whose parents would not allow smoking at all. The perceived parental permissive norms toward alcohol use were associated to a similar higher probability of illicit drug use. The associations were consistently stronger for boys than for girls irrespective of the substance-specific permissiveness (Table 2).

**Table 2:** Adjusted prevalence odds ratios of the effect of parental permissiveness to smoke cigarettes and use alcohol on adolescent illicit drug use at follow-up, by gender

	Overall		Boys		Girls	
	APOR (95% CI)	*P* value	APOR (95% CI)	*P* value	APOR (95% CI)	*P* value
Perceived parental permissiveness to smoke cigarettes^a^						
Would not allow at all	1		1		1	
Would not allow at home	**2.06 (1.43–2.95)**	** < 0.001**	**2.67 (1.68–4.24)**	** < 0.001**	1.38 (0.74–2.57)	0.311
Would allow	**2.17 (1.38–3.40)**	**0.001**	**2.12 (1.21–3.73)**	**0.009**	2.08 (0.96–4.53)	0.064
Would allow/would not allow at home	**2.09 (1.54–2.85)**	** < 0.001**	**2.44 (1.65–3.61)**	** < 0.001**	1.59 (0.94–2.67)	0.081
Do not know	1.19 (0.76–1.86)	0.453	1.56 (0.89–2.74)	0.123	0.71 (0.32–1.58)	0.405
Perceived parental permissiveness to use alcohol^b^						
Would not allow at all	1		1		1	
Would not allow at home	**2.55 (1.78–3.67)**	** < 0.001**	**3.17 (1.99–5.04)**	** < 0.001**	1.80 (0.96–3.35)	0.066
Would allow	**2.01 (1.37–2.95)**	** < 0.001**	**2.23 (1.37–3.63)**	**0.001**	1.72 (0.90–3.28)	0.101
Would allow/would not allow at home	**2.28 (1.69–3.08)**	** < 0.001**	**2.68 (1.82–3.95)**	** < 0.001**	**1.76 (1.06–2.91)**	**0.028**
Do not know	1.21 (0.84–1.75)	0.297	1.14 (0.70–1.83)	0.601	1.29 (0.73–2.28)	0.381

The effects of perceived parental permissiveness to cigarette and alcohol use were examined in separate models. Multilevel mixed-effect logistic regression models with 3 levels (country, school and student) adjusted for gender, age, family composition and parental cigarette smoking

Results in bold are statistically significant at *P* < 0.05

*APOR* adjusted prevalence odds ratios

^a^*n* = 3075 pupils on overall, 1566 boys and 1509 girls

^b^*n* = 3064 pupils on overall, 1562 boys and 1502 girls

Restricting the analysis to baseline non-users, the perceived parental permissiveness toward cigarettes (RR 1.67, 95% CI 1.13–2.48) and alcohol (RR 2.29, 95% CI 1.58–3.32) predicted the onset of illicit drug use.

**Fig. 1:**
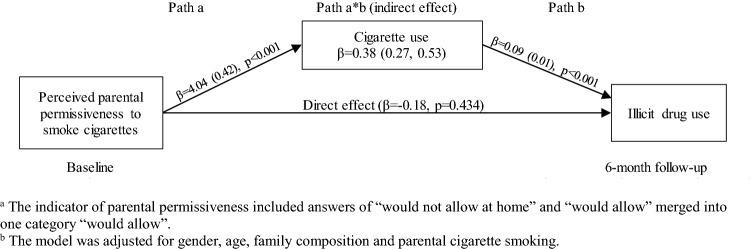
Mediation effect of parental permissiveness to smoke cigarettes on adolescent illicit drug use through cigarette use^a,b^

**Fig. 2:**
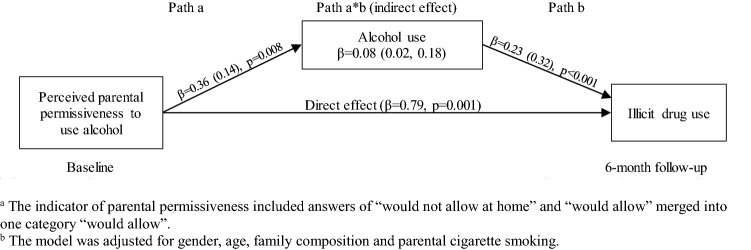
Mediation effect of parental permissiveness to use alcohol on adolescent illicit drug use through alcohol use^a,b^

Adolescent’s own cigarette and alcohol use mediated the effect of perceived parental permissive norms on the risk of using illicit drugs. The perceived parental permissive norms toward cigarettes and alcohol predicted adolescents’ own cigarette and alcohol use (path a), respectively, which in turn were associated with an increased risk of illicit drug use (path b). In case of parental norms toward cigarettes, the association with illicit drug use was fully mediated by adolescent’s cigarette use, and the direct effect was no longer significant (*β* = − 0.18, *p* = 0.434) (Fig. 1). In case of parental norms toward alcohol, the association with illicit drug use was partially mediated by adolescent’s alcohol use, and the direct effect remained significant (*β* = 0.79, *p* = 0.001) (Fig. 2).

## Discussion

This study addresses the relatively novel question of cross-relations between parental permissiveness toward cigarettes and alcohol and the use of illicit drugs among their offsprings. We found that adolescents who perceived permissive parental norms toward cigarette smoking or alcohol use had twice the risk of being users of illicit drugs at follow-up compared to adolescents who perceived completely restrictive norms. The magnitude of the effect is consistent with previous findings on the association between parental permissiveness toward alcohol use and adolescent marijuana and cigarette use [16, 28, 29]. Few other studies found similar results, however, limited to the effect of parental permissive norms toward cigarette and alcohol on the use of the same substance [3, 12, 13, 15, 17].

Li et al. [38] reported that parental cigarette and marijuana use predicted adolescents' use of different substances suggesting that the modeling influence of parental behaviors and norms is not unique to a specific substance [38]. In fact, adolescents may expect their parents to be consistent in setting the norms and perceive parental cigarette and alcohol rules as generalized to other substances. Parental permissive norms toward cigarettes and alcohol can facilitate experimentation with cigarettes and alcohol, that often precedes the use of illicit substances. Indeed, Koning et al. [29] observed that parental restrictive rules toward alcohol may prevent adolescents from involvement in cigarette and cannabis use through their lower engagement in alcohol use [29]. Consistently, we found that adolescents' own cigarette and alcohol use mediate the relationship between parental permissive norms and the risk of illicit drug use. In line with this findings, the use of illicit drugs among adolescents of permissive parents may be related to the use of cigarettes and alcohol.

Parental permissive attitudes favor pro-drinking beliefs and weaken anti-drug norms in adolescence, increasing the risk of engagement in substance use [16, 39, 40]. It has been also observed that adolescents tend to affiliate with peers who hold attitudes and behaviors similar to those of parents [5]. Therefore, perceived parental permissiveness may also favor the affiliation with substance using peers, and/or reinforce the pro-drug influence of peers [3, 7, 9, 14, 17, 22, 40].

The effect of parental alcohol-related norms on the risk of illicit drug use was not fully mediated by adolescents’ use of alcohol. This could be due to a residual independent effect of parental alcohol norms, and/or could be related to adolescents' socialization with peers using licit and illicit substances [5]. This could provide them with opportunities to experiment with substances other than alcohol and cigarettes. In fact, the perceived parental norms toward alcohol had a stronger effect on adolescent's illicit drug use than permissive norms toward cigarettes, and had a stronger impact on the onset of drug use.

The prevalence of illicit drug use was higher among boys (12.0%) than girls (6.4%), consistently with results of the European surveys [1, 2]. According to the ESPAD survey, the prevalence of lifetime any illicit drug use among European teenagers was higher among boys than girls (19% vs. 14%) [2]. Similarly, HBSC reported higher proportion of recent cannabis use in boys over girls, 8% vs. 5%, respectively [1]. In the present study, the direction and magnitude of the effect of perceived parental permissiveness toward cigarette and alcohol use on illicit drug use were consistently stronger among boys. In addition, a tendency to stronger associations among boys was noted even when parental restriction was confined to the domestic environment. Cross-sectional studies generally found significant associations between parental attitudes and their children's substance use independently by gender [5, 16, 19, 24]. However, the perception of parental disapproval toward alcohol acted as a stronger predictor of alcohol use in boys than girls in the Australian longitudinal study by Kelly et al. [32], and a study conducted in the US reported a stronger association of parental anti-drug norms with marijuana use for boys compared to girls [41]. Our findings can be explained by boys' earlier involvement in cannabis use experimentation than girls [1], but also by the fact that a greater proportion of boys than of girls perceived their parents would allow cigarette and alcohol use, possibly indicating stricter parental monitoring of girls compared to boys [42, 43].

Parental banning of substance use only at home and poor communication about the negative effects of substance use, together with substance use behaviors by parents may intensify adolescents' proneness to the same behavior [44]. On the contrary, clear and comprehensive communication may increase the accurate perceptions of parental sanctions and disapproving norms toward drugs, protecting against the involvement in risk behaviors [45–47]. Effective messages of parental disapproval may prevent escalation of substance use also among adolescents who already experiemented drugs [7].

In the present study, the adolescents’ perception of their parents' permissive norms toward legal substances was used as indicator of parental permissiveness. This choice may be questioned in the light of a possible discrepancy between perceived and actual norms. However, previous studies found that offspring's perceptions of parental norms and attitudes were stronger predictors of their substance use behaviors than parental actual norms [46, 48]. Nelson et al. [46] found that adolescents perceive higher parental approval of cigarettes and alcohol use compared to their real attitudes, i.e., adolescents are prone to overestimate the pro-substance norms imposed by parents [46].

This study has several strengths, first and foremost the longitudinal design minimizing the risk of reverse causality. Geographically diverse samples from seven European countries were included in the study, therefore, allowing the study of potentially different family norms and contexts. The surveys were conducted according to a standardized protocol and a standardized questionnaire, reducing possible cross-country misclassification related to data collection. In the statistical analysis, we adopted an approach respectful of the “non-independence” of the individual reports according to higher order clustering (country, school and student). The information accrued in the survey was very comprehensive, allowing the adjustment for several potential confounding factors, and mediation analysis.

The study had also some limitations. It is possible that some unmeasured confounders, such as parental alcohol and cannabis use, and parental norms toward cannabis, affected the relationship between exposure and outcome. All information in this study was self-reported, raising the question of its reliability; however, the anonymous administration of the questionnaire is likely to have attenuated this risk. The results conducted on gender subsamples and on non-users of illicit drugs at baseline could increase the risk of chance error because of the limited sample size.

In conclusion, perceived parental permissiveness toward the use of licit drugs such as cigarette and alcohol predicted adolescents’ use of illicit drugs, especially among boys. Parental norms toward legal drugs may be perceived by their offspring as applying to illicit drugs as well. Parents should be made aware of the importance of norm setting and supported in conveying clear messages of disapproval of all substances, as it may affect the likelihood of their children’s initiation and progression of illicit drug use. The enforcement of parental disapproval, as well as clear and consistent communication about their expectations and sanctions toward cigarette and alcohol use, should be tackled in prevention interventions focused on strengthening parental skills.

## Data Availability

The dataset used for the analyses includes 7079 records of anonymous questionnaires filled by 12- to 14-year-old students in frame of European Drug Addiction Prevention (EU-Dap) trial; the data include information on sociodemographic characteristics; school performance; substance use (cigarettes, alcohol and drug use lifetime and in the last 30 days); knowledge, beliefs, risk perceptions and attitudes toward drugs; self-esteem, decision-making skills, refusal skills; perception of friends’ substance use; parental cigarette use and parental permissiveness toward cigarettes and alcohol. Data are available under request.
